# GMODWeb: a web framework for the generic model organism database

**DOI:** 10.1186/gb-2008-9-6-r102

**Published:** 2008-06-20

**Authors:** Brian D O'Connor, Allen Day, Scott Cain, Olivier Arnaiz, Linda Sperling, Lincoln D Stein

**Affiliations:** 1 Department of Human Genetics, David Geffen School of Medicine, University of California, Los Angeles, California, USA; 2 Cold Spring Harbor Laboratory, Cold Spring Harbor, New York, USA; 3 Centre de Genetique Moleculaire, CNRS, 91198 Gif-sur-Yvette CEDEX, France

## Abstract

The Generic Model Organism Database (GMOD) initiative provides species-agnostic data models and software tools for representing curated model organism data. Here we describe GMODWeb, a GMOD project designed to speed the development of model organism database (MOD) websites. Sites created with GMODWeb provide integration with other GMOD tools and allow users to browse and search through a variety of data types. GMODWeb was built using the open source Turnkey web framework and is available from .

## Abstract 2

GMODWeb is a software framework designed to speed the development of websites for model organism databases.

## Rationale

Model organism databases (MODs) are built around the information needs of scientists working on a single model organism or group of closely related organisms. Examples of MODs include Flybase [1,2], Wormbase [3,4], the Mouse Genome Informatics Database [5,6], the *Saccharomyces *Genome Database [7,8], Gramene, a monocot genomics database [9,10], and ParameciumDB [11,12]. MODs provide scientists with access to information about genomic structure, phenotypes, and mutations along with large-scale datasets such as those generated by gene microarray experiments, single nucleotide polymorphism analyses, or protein-protein interaction studies. A key concern for any MOD is to provide well-designed and convenient community tools for accessing this information. All MODs create databases and website front-ends to fulfill these needs, but a fully functional MOD website is an expensive and time-consuming prospect. As many more model organisms are sequenced the costs, in terms of both time and funds, of independently developing schemata and web-based tools will become prohibitive.

Recognizing this duplication of work, the NIH and the USDA Agricultural Research Service funded the Generic Model Organism Database (GMOD) project with the goal of developing flexible applications that can be used across all MODs. The result is a collection of database and web tools that can be mixed and matched to meet the requirements of new MODs. To date, this effort has produced several high-profile components. A generic and modular relational database schema, called Chado [13], provides the core mechanism to store genomic features, information on gene function, genomic diversity data, literature references, and other common data types. Other popular GMOD tools include Apollo [14], an application for genomic curation, GBrowse [15], a web-based genomic browser that can effectively display genomic features across megabases of sequence, and Textpresso [16], a web tool for literature archiving and searching. While several solutions exist for representing genome annotation data on the web, such as Ensembl [17] and the UCSC Genome Browser [18], no solution exists for representing the full variety of data types needed for a MOD. In this paper we describe GMODWeb, a flexible and extensible framework for creating a MOD website that integrates with other GMOD tools and accommodates many of the data types needed for a model organism database.

## GMODWeb architecture

GMODWeb is based on the Turnkey website generation and rendering framework [19]. Specifically, GMODWeb is a website generated by Turnkey using the GMOD Chado database schema and a series of customizations geared towards MOD communities. GMODWeb provides a starting point for MODs to create websites built on top of GMOD tools and other web-based, bioinformatics applications. Turnkey consists of two distinct components. The first is a code creation tool (Turnkey::Generate) that produces a model view controller (MVC)-based website given a database schema file [20]. The second component (Turnkey::Render) is a page-rendering module that links the generated MVC code to an Apache webserver [21]. This portion of the Turnkey framework uses a collection of open source Perl modules and the popular mod_perl webserver plugin [22]. Each Turnkey component is used in a different phase of website construction. While the MVC generator automates the creation of most site code, the page-rendering module handles the response to user requests received by the webserver.

**Figure 1:**
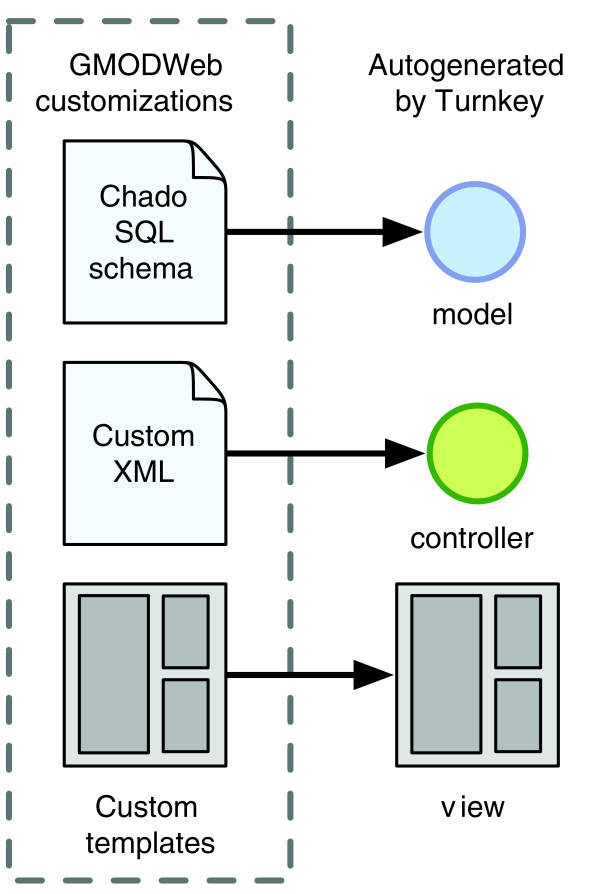
Relationship between GMODWeb and Turnkey. GMODWeb is the result of customizations to a Turnkey website built with the Chado schema. The GMODWeb skin was the product of modifications mainly to the view layer. This included changes to the template view layer, including overriding default templates and CSS changes. Enhancements were also performed with layout changes through controller XML file modifications.

Turnkey-produced websites are strictly divided into MVC layers. This style of abstraction is a useful tool for organizing a web application into manageable layers and improves the overall organization of the software. Likewise, the active code generation approach used by Turnkey, which is similar to the Object Management Group's [23] Model Driven Architecture (MDA) proposal, is especially useful for the GMODWeb project because underlying changes in the data model are quickly and easily integrated into the application [24]. For example, the inclusion of new database modules in Chado can be easily accommodated by regenerating the Turnkey-base site from the Chado database schema file. GMODWeb is produced by simply applying customizations, a GMODWeb 'skin', to this auto-generated site. This decoupling of user interface customization from underlying data structure changes makes the GMODWeb application easy to extend, customize, and maintain. Figure 1 shows the close relationship between GMODWeb and Turnkey.

## GMODWeb site generation and rendering

The creation of a Turnkey site, such as GMODWeb, begins with a SQL schema file used to define the tables in a database and how they relate to each other. This file is abstracted into relationships between objects forming a directed graph. Turnkey::Generate uses the Perl module SQL::Translator to perform the conversion from a SQL schema file to a directed graph object model [25]. For example, in the Chado schema a feature table stores information about genomic features such as mRNAs or genes. This table is linked to many other tables, such as the synonym table via the feature synonym table. The Turnkey::Generate script creates objects representing each table (feature, synonym and feature synonym) and their individual data fields. It then creates links between these objects to mimic the relationships encoded by the schema, in this case linking the feature and synonym tables. A similar process is followed for other table objects.

**Figure 2:**
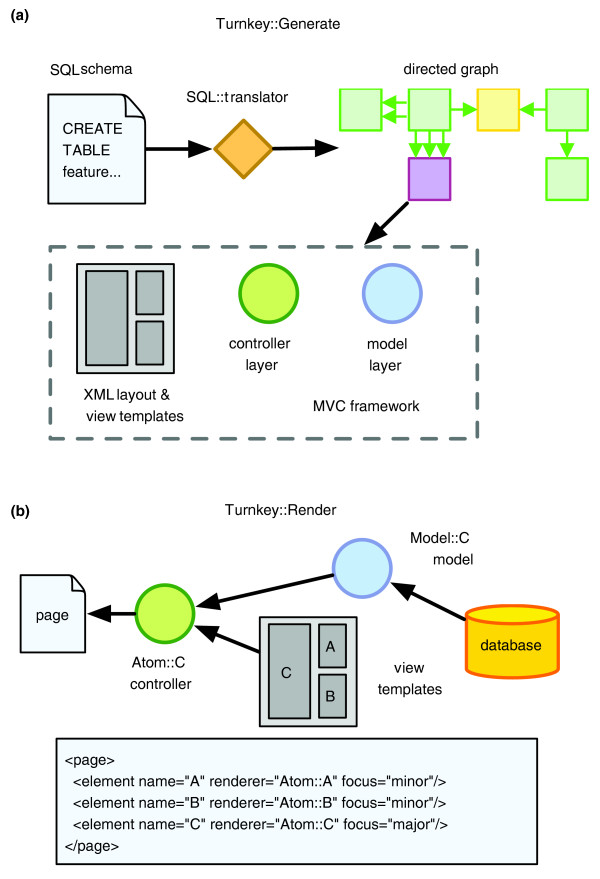
Turnkey::Generate and Turnkey::Render processes. **(a) **The process of creating a Turnkey-based website via Turnkey::Generate is shown. A SQL schema file is processed using SQL::Translator to create a directed graph representation of the relationships between tables. These are used by Turnkey::Generate to create an MVC-based web application. **(b) **The rendering of a Turnkey page by Turnkey::Render is shown. When a client request is received an XML document describing the relationships between objects is consulted. Model objects are created and combined with templates by the atom controller layer to produce a rendered page. This is returned to the client.

Using the relationships encoded by the directed graph, Turnkey::Generate produces an MVC framework, with each layer created using Template Toolkit templates [26]. The model layer, which handles the flow of information to and from the underlying database, is created using a template to produce Class::DBI-based objects [27]. Class::DBI is a convenient tool to connect and retrieve information from the database because it abstracts complex SQL queries into easy-to-use object calls. Controller objects, called atoms in the Turnkey framework, wrap the model objects and provide an abstraction between the view and model objects. They also include the logic necessary to bring these two layers together. The view layer is implemented in Template Toolkit and uses HTML with embedded tags to extract information from controller objects for display to the end user. Turnkey::Generate also creates the Turnkey.xml controller document that describes how model and view objects are to be combined by the atom controller objects. Figure 2a illustrates the MVC-based architecture created with the Turnkey::Generate software. In a typical Perl web application, these MVC layers are usually written by hand in a time-consuming and error prone process. The automatic creation of these objects by Turnkey greatly simplifies the creation of database driven websites.

Once created, the output of Turnkey::Generate is configured to work in an Apache server using the mod_perl framework. The process of rendering a page is handled by Turnkey::Render. When a user requests a certain URL, the Turnkey.xml document is examined by Turnkey::Render and the appropriate Class::DBI model and controller atom objects are instantiated. For example, the feature table described previously has an entry in this XML linking it to the synonym table through the feature synonym table. This provides Turnkey::Render with enough information to create atom and model objects for both the feature and synonym tables. Following this, the appropriate template view objects are created and Turnkey::Render uses the atom controller objects to hand off objects and template files to the Template Toolkit engine for rendering. The resulting HTML output is then returned to the client (Figure 2b).

## GMODWeb customization

Customization is an important ability that all MODs require in their web interfaces. To accommodate this, key design features were integrated into the Turnkey framework to allow for modification of both the site generation and page rendering processes. These include template customization through overriding and cascading style sheet (CSS)-based layouts [28]. Since Turnkey automates the creation of the model, controller, and default view components, customization of templates and CSS documents are where the majority of time is spent adapting GMODWeb to a new MOD (or building a totally new web application from a different schema entirely).

Template overriding provides the ability for MOD developers to create a customized look and feel for a given type of information being displayed in a GMODWeb site. For example, the default genome feature page in GMODWeb is overridden with a custom template that shows a GBrowse-generated image of the feature and its genomic environment if the feature has a genomic location, as is the case for a gene or an mRNA. Various customized templates were also created in GMODWeb for other types of biological objects. These templates are a mixture of plain HTML and Template Toolkit syntax, which is a simplified template language written in Perl but requiring no Perl experience to use. Since most of the customization of a site takes place at this level, the choice of a simple to use but powerful template language was key in allowing non-programmers to customize and create MOD websites.

In addition to template customization, Turnkey-based sites make heavy use of CSS. In the case of GMODWeb, this allows a MOD developer to dramatically change the look and feel of the entire site. Not only can colors and fonts be changed, but element layouts can be reordered. A combination of these customizations, both on the template and CSS levels, can be grouped together into a 'skin' that can easily be parameterized and switched on the fly. This makes it possible for a MOD website to be context-dependent and support a 'print' view or completely different color scheme with the same underlying website and database. For example, a clade-oriented database that provides information on 12 different beetle species could apply a different page color to each species to avoid user confusion.

In most MDA web frameworks these custom templates and CSS documents would normally be overwritten when the website is regenerated. Turnkey, however, allows site designers to create modifications that persist across updates. In this framework, customized templates and CSS documents are placed in a distinct path (a 'skin' directory) that is not overwritten in subsequent rebuilds of the site. So, for example, if the Chado schema underlying GMODWeb was updated, a given MOD could regenerate the GMODWeb site while retaining the customized templates. Changes to existing database tables may result in necessary updates to customized templates but typically the stability of the Chado schema makes this rare for GMODWeb in particular.

Demonstration GMODWeb sites have been created for *Homo sapiens *and *Saccharomyces cerevisiae *and include the basic functionality associated with a typical MOD's homepage. These sites illustrate the common layout for a Turnkey website and show the effects of a customized GMODWeb skin. The sample websites include the ability to search by genomic biological objects and controlled vocabulary terms indexed from the underlying Chado database using the open source search engine Lucene [29]. It was important to be able to query both data types since many types of data in Chado are annotated and linked together through controlled vocabulary terms using various ontologies, such as the Gene Ontology (GO) [30]. Search results will take an end user to either a genomic feature or controlled vocabulary term page rendered using customized GMODWeb templates.

**Figure 3:**
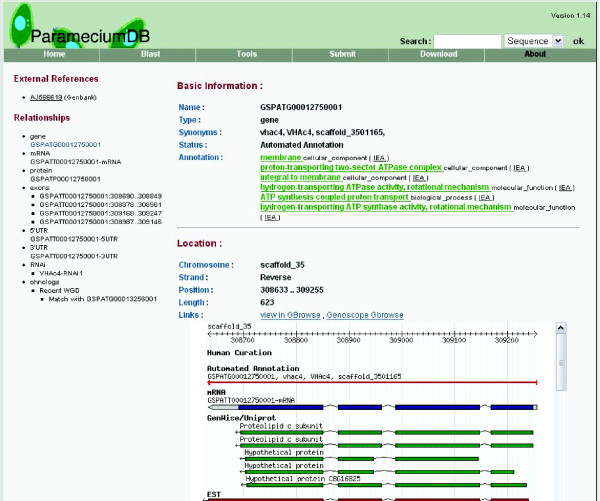
GMODWeb-built ParameciumDB website. An example gene feature rendered with the customized ParameciumDB skin.

Browsing genomic objects reveals several customizations to the default templates. Figure 3 shows a typical gene page using the GMODWeb skin from the ParameciumDB MOD website. In this example, the basic layout of a Turnkey page is evident: the item being rendered, in this case a row from the feature table, is present as the major content panel while linked tables are represented as minor panels on the left-hand side. For this gene feature, two types of linked data were presented on the left: external references (via the feature_dbxref table) and relationships to other features in the database (via the feature_relationship table). Customizations of links and panel headings in both the major and minor panels are shown in this example as well. Similar customization has been applied to other biological objects rendered with GMODWeb.

Further customization was used in the major panel to organize information about the gene feature in an intuitive and helpful way. Related content, such as GO term annotations, genomic location, synonyms, and other information, was included as a summary. The Turnkey framework's flexibility allows custom template authors to easily extract this information using the underlying Class::DBI model objects. In this customized template, simple method calls on the model object were used to extract linked information such as synonyms. Together these modifications have created a gene page that can be leveraged across MODs and provide many of the key pieces of information about biological objects that end users require. Other customized pages render data objects of different types, such as strains or publications. Turnkey pages also contain an edit link that provides a limited but useful facility for editing record data. Authentication is provided by standard HTTP access controls in Apache.

## GMODWeb integration

The example in Figure 3 shows how GMODWeb's templates can be directly integrated with other GMOD projects. In this page, a GBrowse instance was embedded and provided not only a graphical view of the genomic neighborhood but also linked out to nearby genes and other annotations. In addition to GBrowse, the sample GMODWeb sites for *H. sapiens *and *S. cerevisiae *include integration with Textpresso for literature tracking, BlastGraphic for performing Blast analysis, and AmiGO for controlled vocabulary term visualization. These dependencies, which are available from the Biopackages software repository, have been pre-configured to work with the GMODWeb demonstration sites. Packaging the sample applications and their dependencies makes installation and configuration a quick and easy task for site developers and jump-starts the process of setting up new MOD websites. New GMOD components are 'plugged in' to GMODWeb using simple template toolkit and HTML syntax. Essentially, MOD developers creating new GMODWeb sites can treat the customized template files like a 'mash-up' of various web tools using a variety of techniques such as HTML iframes, Template Toolkit includes, and JavaScript.

In addition to web interfaces, GMODWeb also provides Simple Object Access Protocol (SOAP) bindings for accessing data in an automated, programmatic way [31]. This web services approach is designed to allow savvy end users to interact directly with the underlying GMOD Chado database, affording bulk access to features contained within the database. Providing this tool for GMODWeb's model objects makes data access platform agnostic so developers can interact with the service using the language of their choice. Apache2::SOAP was used to bind Class::DBI-based model objects to a SOAP interface [32]. Unlike XML genome feature annotation services, such as the Distributed Annotation System [33], the SOAP bindings present low-level interfaces to database tables. This SOAP interface is pre-configured and immediately available for all MOD sites based on GMODWeb.

## Case study: creating a new MOD website with GMODWeb and Turnkey

*Paramecium*, a unicellular eukaryote that belongs to the ciliate phylum, has served as a genetic model organism for over half a century and is also widely used to teach biology. The genome of *Paramecium tetraurelia *was recently sequenced and annotated at the Genoscope French National Sequencing Center [34]. In anticipation of public release of the data from the sequencing initiative, a project was started in 2005 to develop a *Paramecium *community MOD, ParameciumDB. Its immediate objectives were to integrate the genome sequence and annotations with available genetic data and coordinate the manual curation of the gene models by members of the research community. Ultimately, ParameciumDB should provide a useful resource for the classroom as well.

GMOD's Chado database schema was well suited for this project because of its genetic module, which ensured the integration of both the genetic and sequence data, and its support for describing phenotypes using controlled vocabulary terms. Another important factor in choosing the GMOD toolkit for ParameciumDB was the availability of Turnkey and GMODWeb to generate the MOD's website, since it was anticipated that this would be the most difficult part of the project.

GMODWeb was first tested on a generic installation of Chado, populated with published data from a previously sequenced and annotated *Paramecium *chromosome [35]. The next step was modeling the genetic data, which involved writing a stock module to make it possible to incorporate data about *Paramecium *stock collections. Since the Chado database schema was modified, Turnkey::Generate was used to create a custom website for ParameciumDB.

The last and most time-consuming step for building ParameciumDB was customization of the auto-generated website layout. The overall design of the site components (header, footer, feature page, and so on) was achieved using templates and CSS modifications of the GMODWeb skin, resulting in a custom ParameciumDB skin. Within the auto-generated view code, the feature-relationship atom object was modified to make it possible to recover the complete hierarchy of relationships (for example, gene → mRNA → exon) from the top-level gene feature, even if the feature page being rendered concerned a feature type lower in the hierarchy. Additional static content was added, including a help section, project documentation, and announcements. Finally, commonly used applications were integrated into the ParameciumDB by linking to other bioinformatics tools, such as NCBI's BLAST tool [36], and to forms for data submission by the community.

The templates for pages within ParameciumDB were customized using many of the ideas taken from the GMODWeb sample sites and, in particular, the layout of the sample gene page. The elegance of the Turnkey-based MVC site was most apparent at this level: customization of ParameciumDB was focused on the template view layer while relatively few changes were required to either the model or controller objects. The bulk of the code produced for ParameciumDB was automatically generated and untouched by the customization process. This freed developers to work on the effective presentation of MOD data rather than low level database access or website rendering code. A single developer, working over a period of a few months, brought ParameciumDB to its first release.

The speed with which ParameciumDB was constructed with very limited development resources was a direct result of the many hours spent creating the Turnkey framework and the GMODWeb examples. This effort included the creation of the auto-generation scripts (Turnkey::Generate) that parsed SQL schema files to create object models that accurately represented the underlying data and relationships, templates that used this information to output a mod_perl-based, MVC website (Turnkey::Render), and sample sites using the Chado schema and integrated with other GMOD tools (GMODWeb). In addition, many hours were spent documenting and packaging the Turnkey code and GMODWeb examples to make them easy to install and use as a basis for new development. At least 250 hours of developer time were used to bring the Turnkey to its current, stable release.

For users of the Turnkey framework and GMODWeb, this translates to approximately 1.5 months of full time developer work saved per project using GMODWeb as a starting point for MOD site creation. This estimate has been supported by developers at the ParameciumDB and other locations, including the Clemson University Genomics Institute (CUGI). CUGI is currently using Chado, Turnkey and GMODWeb to speed the development of The Marine Genomics Project (developed in collaboration with the Marine Genomics Group at the Hollings Marine Lab, a NOAA research facility) and the Fagaceae Genomics Web (NSF-PGRP 0605135) [37,38]. The bioinformatics staff at CUGI often juggle several projects at once. Turnkey, the GMODWeb sample sites, and Chado enabled the group to maintain their workload and develop both sites within a three to four month period with only two staff working part time on both projects. The group was able to dedicate primary focus on template design, data collection and new tool development rather than database and web development. As a result, the group now anticipates that it can develop fully functional, manageable GMODWeb and Chado-based websites for its clients and research endeavors in just a few short months, saving money and time (Stephen Ficklin, personal communication). Maintenance of these sites is similarly streamlined since the underlying database can change with a corresponding update to the websites happening in a largely automated fashion while still preserving all customizations.

## Community resources

Turnkey and GMODWeb are both available as source code files from the Turnkey project website and as pre-compiled packages for various Linux distributions from the Biopackages repository for bioinformatics software [19,39]. They are distributed under the GNU public license [40]. All dependencies are provided using the Red Hat Package Manager (RPM) and integration with other programs, such as GBrowse, is accomplished using this same package management system [41]. When a MOD installs GMODWeb via RPMs, a pre-configured GBrowse, Blast server, Textpresso, and other applications are installed and configured to work within GMODWeb immediately. Table 1 shows the software dependencies for GMODWeb, all of which are available either from specific Linux distributions or through Biopackages.

**Table 1:** Software dependencies

Package name	Version	GMOD tool	Description
postgresql-server			The PostgreSQL database server
postgresql	≥7.3		Client libraries for PostgreSQL
perl-Apache-ParseFormData			A Perl library for accessing form data in mod_perl
perl-Class-Base			A Perl base class for other modules
perl-Class-DBI			A Perl tool for abstracting database access
perl-Class-DBI-ConceptSearch			A flexible Perl module for searching databases
perl-Class-DBI-Pager			A Perl tool for breaking database query results into pages
perl-Class-DBI-Pg			A PostgreSQL driver for Class::DBI
perl-Class-DBI-Plugin-Type			A Perl tool for determining data type information
perl-DBD-Pg			A PostgreSQL driver for Perl
perl-DBI			A generic database interface for Perl
perl-Log-Log4perl			Logging software for Perl applications
perl-SQL-Translator			A Perl tool for translating SQL schema into an object model
perl-Template-Toolkit			A template engine for Perl
perl-XML-LibXML			An XML parsing library for Perl
perl-Lucene			A Lucene search engine interface for Perl
perl-Apache2-SOAP			Automatic SOAP bindings for mod_perl
perl-Cache-Cache			A Perl tool used to cache web pages in GMOD-Web
httpd			The Apache webserver
mod_perl	≥2.0.1		A plugin for Apache that executes Perl code
perl			An interpreted language used throughout the Turnkey/GMODWeb project
gbrowse		Yes	A genome feature browser web application from the GMOD project
textpresso		Yes	A literature search web application from the GMOD project
AmiGO		Yes	An ontology browser web application from the GMOD project
chado		Yes	A sample Chado database from the GMOD project
chado-schema		Yes	The Chado schema from the GMOD project
gmod-web	≥1.3	Yes	A GMODWeb site generated with Turnkey for the Chado schema
turnkey	≥1.3		The website generation tool used to create GMODWeb

The GMODWeb application has many software dependencies. This table shows the immediate GMODWeb and Turnkey dependencies on the Fedora Core 2 Linux distribution. All dependencies are available from the Biopackages software repository [39] or are provided by the underlying operating system

The open source nature of GMODWeb and the Turnkey framework provide additional benefits beyond free availability. The GMODWeb project is supported with help from the larger open source development community. The project has benefited immensely from the open source model since its inception. User feedback and contributions to both documentation and the code have resulted in a more robust and user-friendly software tool. Likewise, deployment of GMODWeb-derived websites, in particular ParameciumDB, have identified and resulted in the correction of various performance issues. Information on installation, troubleshooting, and optimization can be found on the Turnkey website [19]. MODs setting up GMODWeb can also solicit help from the email lists either at this site or at GMOD's homepage where the community of users is very active [42].

## Discussion

MODs gather together biological information on a variety of important organisms for the scientific community. A key concern for any MOD is to provide well-designed and easily accessible tools for sharing this information. The GMODWeb project was started to provide a simple and generic solution for quickly creating new MOD websites using the Turnkey framework. GMODWeb, by running directly off of the flexible and extensible Chado schema, can accommodate the wide variety of data types and usage patterns that model organism communities require. GMODWeb offers both a clean MVC framework and pre-built sample websites configured to work with other GMOD tools. Together these features can greatly streamline the process of new MOD website development.

### Benefits and requirements

Developers using GMODWeb and the Turnkey framework tools for creating new MOD websites are released from a variety of tasks that would normally slow a site's creation. These include the automatic generation of several web application layers that are manually created in most other frameworks. Turnkey uses a database schema, or definition, to automatically create most of an MVC framework. For sites based on the Chado schema this means that very little development time is spent building a working website out of individual components. To put this in perspective, the mod_perl API generally is considered a relatively low-level tool for creating websites in Perl. If a MOD developer wanted to create a new website from scratch using Perl and the Apache webserver, they would have to not only create the code to connect to the database (model), control user requests (controller), and generate output back to the user (view) but also an environment to control this flow. Using GMODWeb and, by extension Turnkey, these MVC components along with the rendering engine running in mod_perl and the Apache server have already been written.

What new MOD developers are left with is a focus on template customization, exactly as the case study of the ParameciumDB website illustrated. Since the GMOD Chado schema is available, Turnkey provides the underlying rendering environment and MVC code generation tools, and GMODWeb provides a MOD-focused 'skin', the majority of development time can be spent on customizing a handful of view template files and a CSS document. From a practical perspective this template customization uses plain HTML and simple Template Toolkit syntax to integrate with other web-based tools in straightforward ways. Programmatic access to the underlying data model is also available, making complex queries to the database possible while simple traversals of tables are extremely easy even for inexperienced programmers. In addition, developers building sites with GMODWeb will also spend time adding plain HTML documents, images, and other components to the site. Turnkey allows for these elements to be dropped into place.

### Comparison to other frameworks

Many systems exist for displaying MOD-centric information to end users over the web. These include, for example, the UCSC Genome Browser [18] and Ensembl [17]. In both cases the underlying public web sites can be downloaded and set up locally to mirror the functionality. Generally, these software tools are designed to present information on genomes provided by the host organization and data types specifically supported in the web application. Other tools focus on data integration and querying tasks from a variety of sources. These can include services that index and query datasets for fast searching, such as SRS [43], those that provide a middleware layer that queries across distributed sources, such as BioMoby [44], or those that aggregate data from a variety of sources in a common system, such as Atlas [45], BioWarehouse [46], BIOZON [47], FlyMine [48], or EnsMart [49].

In many ways GMODWeb was designed as a bridge between these different types of MOD tools by providing a generic framework for both browsing and searching model organism data. However, there are several key differences with existing tools. GMODWeb is based on a development framework designed to build websites for specific model organism communities, some of which are not currently served by existing tools such as the UCSC browser. Since Turnkey provides a complete development environment, GMODWeb can be used as a starting point for sophisticated MOD web applications. While GMODWeb is built using the Chado schema, which can model most MOD data and already provides load scripts, the underlying Turnkey tools can also be used to auto-generate websites for other schemata. This makes the integration of other datasets extremely easy since websites for each database can be auto-generated using Turnkey. In contrast, in systems such as FlyMine and other biological data query tools, data must be imported into a common underlying warehouse schema using source definitions that explicitly document the relationships within and between the datasets. Finally, most of the tools compared here are optimized for read only operations. Since GMODWeb includes a full MVC Turnkey framework, applications can be created that include database write back. GMODWeb, for example, includes the ability to update records in a Chado database in addition to browsing and searching them. At their cores, Turnkey and GMODWeb are a development environment and a starting point, respectively, for building cohesive, community driven MOD websites. In contrast, tools such as FlyMine are querying environments geared towards searching across disparate datasets. Each of these projects have distinct, yet complementary, uses.

The Turnkey framework also shares functionality with other generic web development projects. Ruby on Rails [50], Catalyst [51], and Struts/Hibernate [52,53] are just three examples of popular web development frameworks (in Ruby, Perl, and Java, respectively). Each attempts to reduce the time consuming development tasks associated with creating sophisticated web applications. What sets Turnkey apart from other open source solutions is its excellent support for code generation. Tools such as Ruby on Rails and Catalyst include scripts to generate code skeletons but Turnkey is able to use SQL::Translator to create a complete website with only the SQL schema as a starting input. The ability to detect relationships between tables in a database schema and correctly model that in a web application makes Turnkey a valuable generic development tool. In its current release, Turnkey generates its own mod_perl-based web framework. However, the inherent design in no way limits the project to generate exclusively Perl code. In fact, experimental Struts/Hibernate support has been added to the Turnkey project, meaning future version will be able to produce both Perl- and Java-based web applications. In this way, Turnkey does not compete with any of the available website frameworks but acts, instead, as a framework code generation tool.

### Challenges

One of the challenges for any software based on MDA techniques is balancing auto-generated code tied to a particular underlying schema with customized layouts for creating compelling and effective user interfaces. Turnkey, the technology underlying GMODWeb, attempts to solve this limitation by providing a mechanism for bundling specialized skins with an auto-generated website. Since most of the website is automatically created, designers can focus on the quality of the user interface and not on the underlying rendering code. In GMODWeb this translated to extensive customization of the default feature templates. The adoption of GMODWeb by the ParameciumDB project spurred the development of extensive template customizations, which have since been incorporated in the GMODWeb distribution. As additional MODs adopt GMODWeb, we envision the availability of a large library of site-specific customized templates, which can be adopted, altered and expanded by subsequent MOD projects.

As with any open source development project, challenges remain for GMODWeb. Although the project has been available for two years, it has only recently released a 1.0 version. It has been a challenge to attract new MOD users and developers when the project was in this pre-release stage. With the release of ParameciumDB as a proof of concept for GMODWeb in a production environment, the prospects for attracting both new users and developers have improved. Another challenge for the project is the very integrative nature of the GMODWeb application. Since it attempts to bring together several very large web applications, the dependencies for the project are daunting. Maintenance on the various GMOD components integrated into GMODWeb has taken up a large percentage of the development time. However, as a beneficial side effect of this effort, many useful GMOD tools have been packaged as RPMs for distribution through the Biopackages repository, making them available for other projects and uses.

## Conclusion

The rapidity with which ParameciumDB was built, by a very small development team, is encouraging. For this MOD, data modeling was much more time-consuming than building the GMODWeb-based website. In fact, the main difficulty encountered in implementation of ParameciumDB was not with GMODWeb or Turnkey *per se*, but with the installation and tuning of mod_perl and the Apache web server for use in a production environment. The current availability of GMODWeb sample sites and installation dependencies as pre-compiled software packages on Biopackages should make this part of a new project much easier for future MODs.

As the MOD community continues to expand, there will be an increased need to leverage existing tools to store, query, and present MOD data. The GMOD project was created to engineer generic tools to meet these needs. GMODWeb was designed to quickly create MOD websites based on the easy to use, customize, and update Turnkey framework. A GMODWeb MOD site provides not only the ability to browse and search MOD data but it also forms a key link to other tools. As future applications and components become available, GMODWeb will continue to act as a natural point of integration and a central hub for the display of MOD data to end users.

## Abbreviations

CSS, cascading style sheet; CUGI, Clemson University Genomics Institute; GMOD, Generic Model Organism Database; GO, Gene Ontology; MDA, Model Driven Architecture; MOD, model organism database; MVC, model view controller; RPM, RedHat Package Manager; SOAP, Simple Object Access Protocol.

## Authors' contributions

AD and BO designed and wrote the Turnkey framework and GMODWeb. SC, OA, and LS tested and deployed GMODWeb for the ParameciumDB model organism database. LDS is the principal investigator for the Turnkey/GMODWeb project and assisted in the preparation of this report.

## References

[B1] Grumbling G, Strelets V (2006). FlyBase: anatomical data, images and queries.. Nucleic Acids Res.

[B2] Flybase. http://www.flybase.org.

[B3] Schwarz E, Antoshechkin I, Bastiani C, Bieri T, Blasiar D, Canaran P, Chan J, Chen N, Chen W, Davis P, Fiedler T, Girard L, Harris T, Kenny E, Kishore R, Lawson D, Lee R, Mueller H, Nakamura C, Ozersky P, Petcherski A, Rogers A, Spooner W, Tuli M, Van Auken K, Wang D, Durbin R, Spieth J, Stein L, Sternberg P (2006). WormBase: better software, richer content.. Nucleic Acids Res.

[B4] Wormbase. http://www.wormbase.org.

[B5] Blake J, Eppig J, Bult C, Kadin J, Richardson J (2006). The Mouse Genome Database (MGD): updates and enhancements.. Nucleic Acids Res.

[B6] Mouse Genome Informatics Database. http://www.informatics.jax.org.

[B7] Dwight S, Balakrishnan R, Christie K, Costanzo M, Dolinski K, Engel S, Feierbach B, Fisk D, Hirschman J, Hong E, Issel-Tarver L, Nash R, Sethuraman A, Starr B, Theesfeld C, Andrada R, Binkley G, Dong Q, Lane C, Schroeder M, Weng S, Botstein D, Cherry J (2004). *Saccharomyces *genome database: underlying principles and organisation.. Brief Bioinform.

[B8] *Saccharomyces *Genome Database. http://www.yeastgenome.org.

[B9] Jaiswal P, Ni J, Yap I, Ware D, Spooner W, Youens-Clark K, Ren L, Liang C, Zhao W, Ratnapu K, Faga B, Canaran P, Fogleman M, Hebbard C, Avraham S, Schmidt S, Casstevens T, Buckler E, Stein L, McCouch S (2006). Gramene: a bird's eye view of cereal genomes.. Nucleic Acids Res.

[B10] Gramene. http://www.gramene.org.

[B11] Arnaiz O, Cain S, Cohen J, Sperling L (2007). ParameciumDB: a community resource that integrates the *Paramecium tetraurelia *genome sequence with genetic data.. Nucleic Acids Res.

[B12] ParameciumDB. http://paramecium.cgm.cnrs-gif.fr.

[B13] Mungall C, Emmert D, FlyBase Consortium (2007). A Chado case study: an ontology-based modular schema for representing genome-associated biological information.. Bioinformatics.

[B14] Lewis S, Searle S, Harris N, Gibson M, Lyer V, Richter J, Wiel C, Bayraktaroglir L, Birney E, Crosby M, Kaminker J, Matthews B, Prochnik S, Smithy C, Tupy J, Rubin G, Misra S, Mungall C, Clamp M (2002). Apollo: a sequence annotation editor.. Genome Biol.

[B15] Stein L, Mungall C, Shu S, Caudy M, Mangone M, Day A, Nickerson E, Stajich J, Harris T, Arva A, Lewis S (2002). The generic genome browser: a building block for a model organism system database.. Genome Res.

[B16] Mueller H, Kenny E, Sternberg P (2004). Textpresso: an ontology-based information retrieval and extraction system for biological literature.. PLoS Biol.

[B17] Birney E, Andrews D, Caccamo M, Chen Y, Clarke L, Coates G, Cox T, Cunningham F, Curwen V, Cutts T, Down T, Durbin R, Fernandez-Suarez X, Flicek P, Graf S, Hammond M, Herrero J, Howe K, Iyer V, Jekosch K, Kahari A, Kasprzyk A, Keefe D, Kokocinski F, Kulesha E, London D, Longden I, Melsopp C, Meidl P, Overduin B (2006). Ensembl 2006.. Nucleic Acids Res.

[B18] Kent W, Sugnet C, Furey T, Roskin K, Pringle T, Zahler A, Haussler D (2002). The human genome browser at UCSC.. Genome Res.

[B19] Turnkey. http://turnkey.sourceforge.net.

[B20] Gamma E, Helm R, Johnson R, Vlissides J (1995). Design Patterns: Elements of Reusable Object-Oriented Software.

[B21] Apache HTTP Server. http://httpd.apache.org.

[B22] mod_ perl. http://perl.apache.org.

[B23] Object Management Group. http://www.omg.org/mda.

[B24] Frankel D (2003). Model Driven Architecture.

[B25] SQL::Translator. http://sqlfairy.sourceforge.net.

[B26] Template Toolkit. http://template-toolkit.org.

[B27] Class::DBI. http://wiki.class-dbi.com.

[B28] Cascading Style Sheets. http://www.w3.org/Style/CSS.

[B29] Lucene. http://lucene.apache.org.

[B30] Ashburner M, Ball C, Blake J, Botstein D, Butler H, Cherry J, Davis A, Dolinski K, Dwight S, Eppig J, Harris M, Hill D, Issel-Tarver L, Kasarskis A, Lewis S, Matese J, Richardson J, Ringwald M, Rubin G, Sherlock G (2000). Gene Ontology: tool for the unification of biology. The Gene Ontology Consortium.. Nat Genet.

[B31] Simple Object Access Protocol. http://www.w3.org/TR/soap.

[B32] Apache2::SOAP. http://search.cpan.org/~rkobes/Apache2-SOAP-0.72.

[B33] Dowell R, Jokerst R, Day A, Eddy S, Stein L (2001). The distributed annotation system.. BMC Bioinformatics.

[B34] Aury J, Jaillon O, Duret L, Noel B, Jubin C, Porcel B, Segurens B, Daubin V, Anthouard V, Aiach N, Arnaiz O, Billaut A, Beisson J, Blanc I, Bouhouche K, Camara F, Duharcourt S, Guigo R, Gogendeau D, Katinka M, Keller A, Kissmehl R, Klotz C, Koll F, Le Mouel A, Lepere G, Malinsky S, Nowacki M, Nowak J, Plattner H (2006). Global trends of whole-genome duplications revealed by the ciliate *Paramecium tetraurelia*.. Nature.

[B35] Zagulski M, Nowak J, Le Moüel A, Nowacki M, Migdalski A, Gromadka R, Nöel B, Blanc I, Dessen P, Wincker P, Keller A, Cohen J, Meyer E, Sperling L (2004). High coding density on the largest *Paramecium tetraurelia *somatic chromosome.. Curr Biol.

[B36] Altschul S, Gish W, Miller W, Myers E, Lipman D (1990). Basic local alignment search tool.. J Mol Biol.

[B37] Marine Genomics Project. http://www.marinegenomics.org.

[B38] Fagaceae Genomics Web. http://www.fagaceae.org.

[B39] Biopackages.net. http://biopackages.net.

[B40] GNU Public License. http://www.gnu.org/licenses/gpl.txt.

[B41] Red Hat Package Manager. http://www.rpm.org.

[B42] Generic Model Organism Database Project. http://gmod.org.

[B43] Etzold T, Argos P (2001). SRS-an indexing and retrieval tool for flat file data libraries.. Bioinformatics.

[B44] Wilkinson M, Links M (2002). BioMOBY: An open source biological web services proposal.. Brief Bioinform.

[B45] Shah S, Huang Y, Xu T, Yuen M, Ling J, Ouellette B (2005). Atlas - a data warehouse for integrative bioinformatics.. BMC Bioinformatics.

[B46] Lee T, Pouliot Y, Wagner V, Gupta P, Stringer-Calvert D, Tenenbaum J, Karp P (2006). BioWarehouse: a bioinformatics database warehouse toolkit.. BMC Bioinformatics.

[B47] Birkland A, Yona G (2006). BIOZON: a system for unification, management and analysis of heterogeneous biological data.. BMC Bioinformatics.

[B48] Lyne R, Smith R, Rutherford K, Wakeling M, Varley A, Guillier F, Janssens H, Ji W, Mclaren P, North P, Rana D, Riley T, Sullivan J, Watkins X, Woodbridge M, Lilley K, Russell S, Ashburner M, Mizuguchi K, Micklem G (2007). FlyMine: an integrated database for *Drosophila *and *Anopheles *genomics.. Genome Biol.

[B49] Kasprzyk A, Keefe D, Smedley D, London D, Spooner W, Melsopp C, Hammond M, Rocca-Serra P, Cox T, Birney E (2004). EnsMart: a generic system for fast and flexible access to biological data.. Genome Res.

[B50] Ruby on Rails. http://www.rubyonrails.org.

[B51] Catalyst. http://www.catalystframework.org/.

[B52] Struts. http://struts.apache.org.

[B53] Hibernate. http://www.hibernate.org.

